# A systematic review of AI literacy scales

**DOI:** 10.1038/s41539-024-00264-4

**Published:** 2024-08-06

**Authors:** Tomáš Lintner

**Affiliations:** 1 Department of Educational Sciences, Faculty of Arts, Masaryk University, Brno, Czech Republic; 2 Institute SYRI, Brno, Czech Republic

**Keywords:** Education, Education

## Abstract

With the opportunities and challenges stemming from the artificial intelligence developments and its integration into society, AI literacy becomes a key concern. Utilizing quality AI literacy instruments is crucial for understanding and promoting AI literacy development. This systematic review assessed the quality of AI literacy scales using the COSMIN tool aiming to aid researchers in choosing instruments for AI literacy assessment. This review identified 22 studies validating 16 scales targeting various populations including general population, higher education students, secondary education students, and teachers. Overall, the scales demonstrated good structural validity and internal consistency. On the other hand, only a few have been tested for content validity, reliability, construct validity, and responsiveness. None of the scales have been tested for cross-cultural validity and measurement error. Most studies did not report any interpretability indicators and almost none had raw data available. There are 3 performance-based scale available, compared to 13 self-report scales.

## Introduction

The integration of Artificial Intelligence (AI) into various segments of society is increasing. In medicine, AI technologies can facilitate spine surgery procedures^1^, effectively operate healthcare management systems^2,3^, and provide accurate diagnosis based on medical imaging^4^. In education, AI systems contribute to effective teaching methods and enable accurate student assessments^5^. In science, AI plays a role in generating innovative hypotheses, surpassing the creative limits of individual researchers^6^ and aids scientific discovery^7,8^.

With the increasing integration of AI in society, many new AI-related jobs are emerging, and many existing jobs now require AI re-skilling. Job postings requiring skills in machine learning and AI have significantly increased^9,10^. In the U.S., there was a dramatic rise in demand for AI skills from 2010 to 2019, surpassing the demand for general computer skills with AI proficiency providing a significant wage premium^11^. Furthermore, many companies have been reducing hiring in jobs not exposed to AI, suggesting a significant restructuring of the workforce around AI capabilities^12^.

AI’s impact extends beyond the job market; it also alters the way people process information. It has enabled the production of deepfake audiovisual materials unrecognizable from reality with many websites casually offering services of face-swapping, voice-cloning, and deepfake pornography. Consequently, there has been a significant rise in fraud and cyberbullying incidents involving deepfakes^13^. The emergence of deepfakes has also led to a new generation of disinformation in political campaigns^14^. Research shows that people cannot distinguish deepfakes but their confidence in recognizing them is high, which suggests that they are unable to objectively assess their abilities^15,16^.

In the context of AI permeating job market and the spread of deepfakes, AI literacy becomes a key concern. As a recent concept, AI literacy has not yet been firmly conceptualized. AI literacy is often viewed as an advanced form of digital literacy^17^. In its basic definition, AI literacy is the ability to understand, interact with, and critically evaluate AI systems and AI outputs. A review aimed at conceptualizing AI literacy based on the adaptation of classic literacies proposed four aspects crucial for AI literacy—know and understand, use, evaluate, and understanding of ethical issues related to the use of AI^18^. Research and practice differ in specific expectations of AI literacy based on age—most agree that it should be part of education from early childhood education with more complex issues taught in older ages. While some authors argue that technical skills like programming should be a part of AI literacy, most agree it should encompass more generalizable knowledge and interdisciplinary nature^19,20^. Many global initiatives to promote AI literacy are emerging^20^ and AI literacy is becoming a part of the curriculum in early childhood education^21^, K-12 education^22–24^, as well as in higher education^18,19^ in several educational systems. At the same time, however, both researchers and educators pay little attention to development and understanding of instruments to assess AI literacy at different educational levels^22^.

Utilizing quality AI literacy instruments is crucial for understanding and promoting AI literacy development. This systematic review will aim to aid both researchers and educators involved in research and evaluation of level and development of AI literacy. This systematic review has the following objectives:

- to provide a comprehensive overview of available AI literacy scales
- to critically assess the quality of AI literacy scales
- to provide guidance for research which AI literacy scales to use considering the quality of the scales and the context they are suitable for.

## Results

### Overview of AI literacy scales

**Fig. 1:**
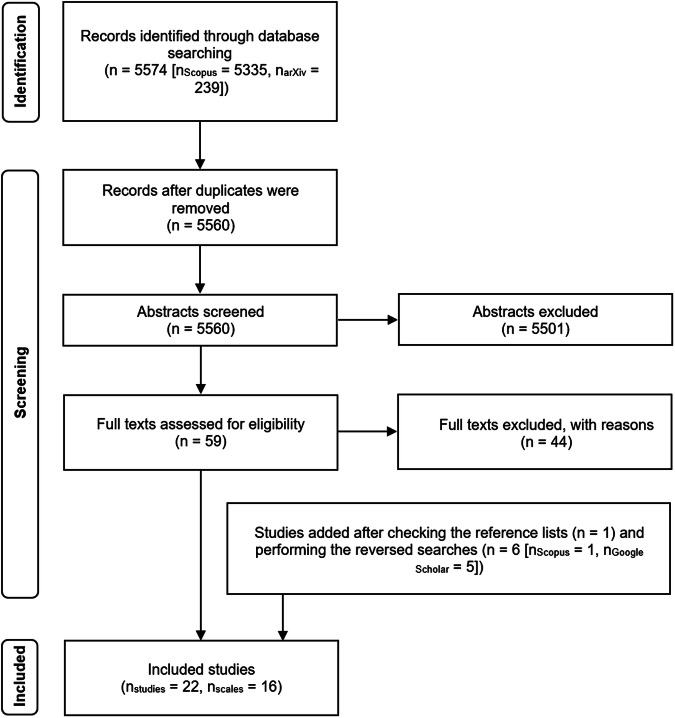
PRISMA flowchart. The PRISMA flowchart shows the study identification procedure.

The initial search yielded 5574 results. After removing duplicate references, a total of 5560 studies remained. Figure 1 presents an overview of the literature search, screening, and selection process. During the initial screening, I manually reviewed titles and abstracts. In this step, I excluded 5501 records, which did not meet the inclusion criteria outlined in *Methods* section. I assessed the full texts of the remaining 59 records for eligibility and I checked their reference lists for other potentially relevant studies. After the full-text screening, I excluded 44 records. Most studies were excluded because they did not perform any scale validation, e.g. ^25–27^ or did not touch upon the concept of AI literacy^28^. AI4KGA^29^ scale was excluded because the author did not provide the full item list and did not respond to my request for it, making it questionable whether the scale can be used by anyone else. While self-efficacy is somewhat a distinct construct from self-reported AI literacy, the distinction between the two is heavily blurred. I therefore decided to adopt a more inclusive approach when assessing the relevancy of the measured constructs and included Morales-García et al.’s GSE-6AI^30^ and Wang & Chuang’s^31^ AI self-efficacy scale as well. I added one publication from the reference lists of the included studies to the final selection and six studies from the reverse searches, yielding a total of 22 studies validating or revalidating 16 scales.

Table 1 presents the studies’ basic descriptions. The included scales share several characteristics. Only a minority of the scales are performance-based^32–34^, with most scales relying on self-assessment-based Likert items^30,31,35–45^. Most scales have multiple factor structures. Constructing AI literacy scales has started only recently as all scales were constructed in the last three years, with the oldest being MAIRS-MS^43^ from 2021. MAIRS-MS^43^, SNAIL^45^, and AILS^36^ are also the only scales to this date, which have been revalidated by another study^46–51^. On the other hand, the scales vary by their target populations. Most of them target general population^31,34,36,42,44–47^ or higher education students^30,32,37–39,43,48–51^, with three of them targeting secondary education students^33,35,41^, and one targeting teachers^40^.

**Table 1:** Scales’ characteristics

basic scale description				validation sample^a^		factor structure	
scale	type	items	target population	N	Age (years)	no. of factors	factor description
AI literacy test^32^	performance-based	31 including 30 multiple-choice items with 4 options each and 1 sorting item	university students	1286	23.6 ± 4.5	1	single factor
AI-CI^33^	performance-based	20 multiple-choice items	middle school students	981, 108	?	1	single factor
AILQ^35^	self-report	32 5-point Likert items	secondary school students	363	13.1 ± 1.4	6 level one factors, 4 level two factors	(F1) affective learning: *(F1a) Intrinsic motivation (F1b) confidence* (F2) behavioural learning: *(F2a) behavioural commitment (F2b)* c*ollaboration* (F3) cognitive learning: *(F3a) know and understand (F3b) evaluate and create* (F4) ethical learning
AILS^36,46,47^	self-report	12 7-point Likert items	general population	325, 402, 536	29.7 ± 7.3, ?, ?	4	(F1) awareness (F2) use (F3) evaluation (F4) ethics
AISES^31^	self-report	22 7-point Likert items	general population	314	?	4	(F1) assistance (F2) anthropomorphic interaction (F3) comfort with AI (F4) technological skills
Chan & Zhou’s EVT based instrument (knwl. of gen. AI subscale)^37^	self-report	5 5-point Likert items	university students	405	29.9 ± ?	1	single factor
ChatGPT literacy scale^38^	self-report	25 5-point Likert items	college students	822	22.7 ± 2.6	5	(F1) technical proficiency (F2) critical evaluation (F3) communication proficiency (F4) creative application (F5) ethical competence
GSE-6AI^30^	self-report	6 4-point Likert items	medical students	469	19.7 ± 2.5	1	single factor
Hwang et al.’s instrument^39^	self-report	19 5-point Likert items	college students	318	?	4	(F1) critical understanding (F2) artificial intelligence social impact recognition (F3) artificial intelligence technology utilization (F4) ethical behaviour
Intelligent TPACK^40^	self-report	29 7-point Likert items	teachers	647	?	5	(F1) technological knowledge (F2) technological pedagogical knowledge (F3) technological content knowledge (F4) technological pedagogical content knowledge (F5) ethics
Kim & Lee’s instrument^41^	self-report	30 5-point Likert items	middle school students	1222	?	6	(F1) societal impact (F2) understanding of AI (F3) AI execution plans (F4) problem solving with AI (F5) data literacy (F6) AI ethics
MAILS^42^	self-report	34 11-point Likert items	adults	300	32.1 ± 11.7	8 level one factors, 4 level two factors	(F1) AI literacy: *(F1a) use & apply AI (F1b) understand AI (F1c) detect AI (F1d) AI ethics* (F2) create AI (F3) AI self-efficacy: *(F3a) AI problem solving (F3b) AI learning* (F4) AI self-competency: *(F4a) AI persuasion literacy (F4b) AI emotion regulation*
MAIRS-MC^43,48^	self-report	22 5-point Likert items	medical students	865, 502	21.3 ± 2.0, 22.7 ± 2.8	4	(F1) cognition (F2) ability (F3) vision (4) ethics
Pinski & Belian’s instrument^44^	self-report	13 7-point Likert items	general population	50	32.8 ± 13.2	5	(F1) AI technology knowledge (F2) human actors in AI knowledge (F3) AI steps knowledge (F4) AI usage experience (F5) AI design experience
SAIL4ALL^34^	performance-based	56 true/false or 5-point Likert items	general population	619 for true/false version, 393 for Likert scale version	45.8 ± 12.2 for true/false version, 46.3 ± 15.4 for Likert scale version	4 themes (subscales) – each with 1–2 factors	(T1) what is AI? (T2) what can AI do? (T3) how does AI work? (T4) how should AI be used?
SNAIL^45,49–51^	self-report	31 7-point Likert items	adult non-experts, university students, medical students	415, 25, 653, 377	39.5 ± 13.6, 22.9 ± 2.3, 25.6 ± ?, 22.5 ± 3.2	3	(F1) technical understanding (F2) critical appraisal (F3) practical application

^a^in case the scale has been revalidated, the numbers refer to the original sample followed by the revalidation samples. N = number of participants used for scale development. ? = not reported.

While the authors of the scales drew their conceptualizations of AI literacy from different sources and their scales target different populations, they largely overlap with core competencies comprising AI literacy. By looking at the authors’ conceptualizations of key competencies comprising AI literacy, virtually all scales recognize several core competencies as fundamental to AI literacy. First, they emphasize the technical understanding of AI, distinguishing it from mere general awareness about the technology. Secondly, they consider the societal impact of AI as a critical component. Lastly, AI ethics is acknowledged as an essential aspect. These competencies collectively form the foundational elements of AI literacy, and they are consistently present as factors across the various scales. There is a consensus among the authors of the scales about the three competencies being essential for both secondary and higher education students as well as general population and medical professionals. On the other hand, the authors of the scales differ in perceiving higher-order AI-related skills—creation and evaluation of AI—as components of AI literacy. In the original Ng et al.’s conceptualization^18^, creation and evaluation of AI are core components of AI literacy. MAILS^42^ drawing from the Ng et al.’s conceptualization^18^ identified creation of AI as a related, but separate construct from AI literacy. AILQ^35^, on the other hand, drawing from the same conceptualization includes creating AI as a core part of AI literacy. Several other scales also consider the ability to critically evaluate AI as a core part of AI literacy^32–34,36,38,44^. Considering the widespread integration of AI into daily and professional life, a question arises, whether the skills to create and critically evaluate AI will not have to be included as core competencies of AI literacy in near future, as those competencies might be crucial for functional AI literacy.

### Quality assessment

I assessed the quality of the scales based on the COSMIN^52–56^ measurement properties and additionally on interpretability and feasibility. The Methods section provides a detailed explanation of these individual properties. Table 2 shows quality assessment of the scales based on the COSMIN^52–56^ and GRADE^57^ criteria. Overall, the scales demonstrated good structural validity and internal consistency. On the other hand, only a few have been tested for content validity, reliability, construct validity, and responsiveness. None of the scales have been tested for cross-cultural validity and measurement error. Most studies did not report any interpretability indicators and almost none reported scales’ average completion time (Tables 3 and 4).

**Table 2:** Scales’ quality assessment based on COSMIN and GRADE criteria

	content validity	structural validity	internal consistency	cross-cultural validity	measurement invariance	reliability	measurement error	construct validity	responsiveness
AI literacy test^32^	++	++++	++++	?	?	?	?	++++	?
AI-CI^33^	+++	+++	++++	?	?	?	?	?	++++
AILQ^35^	+++	++++	++++	?	?	?	?	?	+
AILS^36,46,47^	+	++++	++++	?	?	++	?	++++	?
AISES^31^	?	++++	++++	?	?	?	?	?	?
Chan & Zhou’s EVT based instrument (knwl. of gen. AI subscale)^37^	?	++++	++++	?	?	?	?	?	?
ChatGPT literacy scale^38^	+++	++++	++++	?	?	?	?	++++	?
GSE-6AI^30^	?	++++	++++	?	++++	?	?	?	?
Hwang et al.’s instrument^39^	?	++++	++++	?	?	?	?	?	?
Intelligent TPACK^40^	?	++++	++++	?	?	?	?	?	?
Kim & Lee’s instrument^41^	++	++++	++++	?	?	?	?	++++	?
MAILS^42^	?	++++	++++	?	?	?	?	++++	?
MAIRS-MC^43,48^	++	++++	++++	?	++++	?	?	?	?
Pinski & Belian’s instrument^44^	+	+	+++	?	?	?	?	?	?
SAIL4ALL true/false format^34^	?	±	±	?	?	?	?	++++	?
SAIL4ALL Likert scale format^34^	?	±	±	?	?	?	?	++++	?
SNAIL^45,49–51^	?	++++	++++	?	?	+	?	++++	++

**Table 3:** Scales’ interpretability indicators

	score distribution		missing data		floor & ceiling effects	
	M	SD	% of missing items	% of missing total scores	items with >15% responses with lowest score (floor)	items with >15% responses with highest score (ceiling)
AI literacy test^30^	18.8	5.6	?	?	?	?
AILQ^44^	?	?	?	?	?	?
AILS^33,37,38^	?	?	?	?	?	?
AISES^29^	?	?	?	?	?	?
Chan & Zhou’s EVT based instrument (knwl. of gen. AI subscale)^41^	20.1	3.3	?	?	?	?
Hwang et al.’s instrument^42^	?	?	?	?	?	?
Intelligent TPACK^45^	3.4	?	?	?	?	?
Kim & Lee’s instrument^43^	?	?	?	?	?	?
MAILS^39^	4.7	2.6	0.7	0	1 item from AI ethics, 2 items from create AI, 2 items from learning	1 item from AI emotion regulation
MAIRS-MC^31,34^	88.3^31^ 63.1^34^	15.9^31^	?	?	?	?
Pinski & Belian’s instrument^40^	?	?	?	?	?	?
SNAIL^32,35,36^	3.7^32^, 4.2^35^	1.1^32^, 0.9^35^	0.3^32^, 0.2^35^	0^32^, 0^35^	14 items^32^, 12 items^35^	0 items^32^, 0 items^35^

**Table 4:** Scales’ feasibility indicators

	available language(s)	completion time (in minutes)	
		M	SD
AI literacy test [31a]	German, English (not validated)	?	?
AI-CI^33^	English	?	?
AILQ^35^	English	?	?
AILS^36,46,47^	English, Turkish	?	?
AISES^31^	English	?	?
Chan & Zhou’s EVT based instrument (knwl. of gen. AI subscale) [3a]	English	?	?
ChatGPT literacy scale^38^	English	?	?
GSE-6AI^30^	Spanish, English (not validated)	?	?
Hwang et al.’s instrument^39^	English	?	?
Intelligent TPACK^40^	English	?	?
Kim & Lee’s instrument^41^	Korean	?	?
MAILS [4a]	German, English (not validated)	16:05	5:46
MAIRS-MC^43,48^	Turkish, Persian	?	?
Pinski & Belian’s instrument^44^	English	?	?
SAIL4ALL true/false format^34^	English	8:51	5:30
SAIL4ALL Likert scale format^34^	English	9:38	5:52
SNAIL^45,49–51^	English, German, Turkish	5:39^45^, 8:01^49^, 5:52^51^	2:19^45^, 1:53^49^, 2:27^51^

#### AI literacy test^32^

This is a performance-based scale assessing AI-related knowledge through 30 multiple-choice questions, each with a single correct option, and includes one sorting question. The authors used item response theory (IRT) models to confirm the scale’s single-factor structure. The authors drew from Long & Magerko’s^58^ conceptualization of AI literacy, which works with a set of 17 AI competencies grouped into five overarching areas: What is AI?, What can AI do?, How does AI work?, How should AI be used?, and How do people perceive AI?. The authors developed the scale primarily for higher-education students—the scale comprises both items which could be considered specialized advanced knowledge (e.g., distinguishing between supervised and unsupervised learning), but also basic general knowledge (e.g., recognizing areas of daily life where AI is used). However, the scale is arguably also suitable for any professionals who encounter AI in their work. There is some limited evidence for the scale’s content validity and high evidence for the scale’s structural validity, internal consistency, and construct validity. It is currently available in German and English, although English version has not yet been revalidated. It is possible that the content of some questions—especially those dealing with a typical use of AI in practice—will need to be changed in the future due to developments in AI, rendering some of the present items obsolete.

#### AI-CI—AI literacy concept inventory assessment^33^

AI-CI is a performance-based concept inventory scale for middle school students assessing AI-related knowledge through 20 multiple-choice questions. The authors used their own AI literacy curriculum^59^ to design the scale’s content. IRT was used for validation. There is good evidence for the scale’s content validity and structural validity, and high evidence for the scale’s internal consistency and responsiveness. It is currently available in English. The content of the items appears to be more general and less dependent on the context of AI developments compared to the AI literacy test^32^.

#### AILQ—AI literacy questionnaire^35^

AILQ is aimed at secondary education students. The scale employs authors’ conceptualization of cognitive domains of AI literacy stemming from their exploratory review^18^ adding affective, behavioural, and ethical learning domains. The authors employed a CFA which resulted in identification of the scale’s four-factor structure paralleling the four learning domains. There is moderate positive evidence for the scale’s content validity, high positive evidence for the scale’s structural validity and internal consistency, and very low positive evidence for the scale’s responsiveness. It is currently available in English.

#### AILS—AI literacy scale^36^

AILS scale targets general population in the context of human–AI interaction (HAII). The authors drew from their own conceptualization of AI literacy grounded in their literature review resulting in four constructs of AI literacy: awareness, use, evaluation, and ethics. The four constructs are parallel to the scale’s four factors confirmed by a CFA. The scale has since been revalidated in Turkish language^46,47^, however, no direct cross-cultural validation has been performed. There is very low positive evidence for the scale’s content validity, high positive evidence for the scale’s structural validity and internal consistency, low evidence for reliability, and high positive evidence for construct validity.

#### AISES—AI self-efficacy scale (AISES)^31^

AISES is aimed at assessing AI self-efficacy of general population. The scale’s conceptualization is grounded in previous technology-related self-efficacy research^60,61^. A CFA confirmed the scale’s four-factor structure. There is high positive evidence for the scale’s structural validity and internal consistency, however, content validation on the target population was not performed. It is currently available in English.

#### Chan & Zhou’s EVT based instrument for measuring student perceptions of generative AI (knowledge of generative AI subscale)^37^

This subscale is part of a larger instrument aimed at assessing perceptions of generative AI of university students. Here, I reviewed only the subscale dealing with the self-perceived AI literacy. The authors drew from their own conceptualization of AI literacy grounded in their literature review. The items revolve around generative AI’s limitations and potential biases. CFA confirmed the subscale’s single-factor structure. There is high positive evidence for the subscale’s structural validity and internal consistency, however, content validation of the scale is disputable. It is currently available in English.

#### ChatGPT literacy scale^38^

The scale for college students is focused specifically on assessing AI literacy using ChatGPT. The scale is grounded in a Delphi survey performed by the authors. There is good evidence for the scale’s content validity and high evidence for the scale’s structural validity, internal consistency, and construct validity. The scale is available in English language.

#### GSE-6AI—brief version of the general self-efficacy scale for use with artificial intelligence^30^

The scale comprises only six items, making it suitable for a rapid assessment of AI self-efficacy. There is high positive evidence for the scale’s structural validity, internal consistency, and measurement invariance by gender, however, content validation on the target population was not performed. It is currently available in Spanish and English.

#### Hwang et al.’s digital literacy scale in the artificial intelligence era for college students^39^

This scale targets higher education students and the authors also largely drew from Long & Magerko’s^58^ conceptualization of AI literacy. The authors employed a CFA which resulted in identification of the scale’s four-factor structure. There is high positive evidence for the scale’s structural validity and internal consistency, however, content validation on the target population was not performed. It is currently available in English.

#### Intelligent TPACK—technological, pedagogical, and content knowledge scale^40^

Intelligent-TPACK aims to assess teachers’ self-perceived level of AI-related knowledge necessary for integration of AI in their pedagogical work. It draws from the TPACK framework^62^ adding an aspect of AI ethics. The scale assesses teachers’ knowledge of four AII-based tools: Chatbots, intelligent tutoring systems, dashboards, and automated assessment systems arguing that those are the most prevalent AI-based technologies in K-12 education. A CFA showed scale’s five-factor structure comprising the original TPACK dimensions with ethics. There is high positive evidence for the scale’s structural validity and internal consistency, however, content validation on the target population was not performed. It is currently available in English.

#### Kim & Lee’s artificial intelligence literacy scale for middle school students^41^

This scale targets secondary education students. The authors drew from an ad hoc expert group’s conceptualization of AI literacy revolving around AI’s societal impact, understanding of AI, AI execution plans, problem solving, data literacy, and ethics. The authors employed a CFA which resulted in identification of the scale’s six-factor structure. There is some limited positive evidence for the scale’s content validity and high evidence for the scale’s structural validity, internal consistency, and construct validity. So far, the scale is only available in Korean.

#### MAILS—meta AI literacy scale^42^

MAILS is a general-population scale developed from Ng et al.’s^18^ conceptualization of AI literacy with four areas: know and understand AI, use and apply AI, evaluate and create AI, and AI Ethics. Additionally, it includes further psychological competencies related to the use of AI above the Ng et al.’s^18^ areas of AI Literacy—self-efficacy and self-perceived competency. It is the most extensive instruments out of the reviewed instruments. Resulting from a confirmatory factor analysis (CFA), the four AI literacy areas were not found to be all part of a single AI literacy concept—creating AI was found to be a separate factor. The authors made the scale modular in a sense that each of the resulting factors can be measured independently of each other—AI literacy (18 items), create AI (4 items), AI self-efficacy (6 items), and AI self-competency (6 items). There is high positive evidence for the scale’s structural validity, internal consistency, and construct validity, however, content validation on the target population was not performed. It is currently available in German and English, although English version has not yet been revalidated. There is evidence that the scale has good interpretability, although the scale shows some indication of floor effects for five items and ceiling effect for one item. The scale is feasible for a quick assessment of AI literacy, with most participants completing the scale within 20 min.

#### MAIRS-MS—medical artificial intelligence readiness scale for medical students^43^

MAIRS-MS is aimed at medical students and the authors developed it from conceptualization of AI readiness of both professionals and medical students. Originally developed for Turkish medical students, the scale has since been revalidated in Persian language in Iran^48^, however, no direct cross-cultural validation has been performed. CFAs on two samples^43,48^ confirmed the scale’s four-factor structure. There is some limited positive evidence for the scale’s content validity and high evidence for the scale’s structural validity, internal consistency, and invariance by gender.

#### Pinski & Belian’s instrument^44^

This scale targets general population. The authors draw from their own conceptualization of AI literacy grounded in their literature review. The authors employed a structural equation model to come to the scale’s five-factor structure. Due to a limited sample size, there is only limited positive evidence for the scale’s content and structural validity, and medium evidence for internal consistency. It is currently available in English.

#### SAIL4ALL—the scale of artificial intelligence literacy for all^34^

SAIL4ALL is a general-population scale comprising four distinct subscales, which can be used independently. However, the individual subscales cannot be aggregated to get an overall AI literacy score. The scale can also be used in both true/false and Likert-scale format. The authors drew from Long & Magerko’s^58^ conceptualization of AI literacy. Content validation on the target population was not performed. There is mixed evidence for the scale’s structural validity and internal consistency. On the one hand, a two-factor “What is AI?” subscale, a single-factor “How does AI work?”, and a single-factor “How should AI be used?” show good structural validity and internal consistency in both true/false and Likert scale format. On the other hand, “What can AI do?” subscale shows poor structural validity and internal consistency. There is an indication that the scale suffers from the ceiling effect.

#### SNAIL—scale for the assessment of non-experts’ AI literacy^45^

SNAIL is a general-population scale developed from the authors’^63^ extensive Delphi expert study’s conceptualization of AI literacy. The authors used an exploratory factor analysis to assess the scale’s factor structure resulting in a three-factor TUCAPA model of AI literacy—technical understanding, critical appraisal, and practical application. The scale has since been revalidated in Turkish language^50^ and in German language and for the use of learning gains using retrospective-post-assessment^49^, however, no direct cross-cultural validation has been performed. There is high positive evidence for the scale’s structural validity and internal consistency, and due to a small longitudinal sample size, only limited evidence for the scale’s reliability and responsiveness. Content validation on the target population was not performed in any of the four studies^45,49–51^, nor in the Delphi study^63^. There is an indication that the scale suffers from the floor effect, with almost half of the items having >15% responses with the lowest possible score. The scale is feasible for a quick assessment of AI literacy, with most participants completing the scale within 10 min.

## Discussion

This review identified 22 studies (re)validating 16 scales designed to assess AI literacy. Unfortunately, none of the scales showed positive evidence for all COSMIN measurement properties and most studies suffered from poor methodological rigour. Furthermore, the scales’ interpretability and feasibility also remain largely unknown due to most studies not reporting the necessary indicators, and, with an exception of Laupichler et al.^45,49^, not providing open data. By not providing public open data, the authors not only prevent calculations of some of the relevant quality indicators but may also contribute to the replicability crisis in science. Most studies did not report percentages of missing data and strategies they employed to address missing data, which puts their credibility into question.

Considering the overall limited evidence for the quality of the scales, I will formulate recommendations drawing mainly from the COSMIN priorities considering content validity the most important measurement property, the scales’ potential for efficient revalidation, and the target populations.

When aiming for an assessment of general population, AILS^36^ is the scale with the most robust quality evidence. It showed at least some evidence for content validity and reliability, while showing good evidence for structural validity and internal consistency. Also, it has been revalidated in another two studies^46,47^. Pinski & Belian’s instrument^44^ also showed at least some evidence for content validity, but it has been validated on a limited sample, requiring revalidation on a bigger sample in the future. The following general population scales did not include target population in the content validation phase. SNAIL^45^ was constructed on an elaborate Delphi study^63^, it has been revalidated in another three studies^49–51^ including one with comparative self-assessment gains^49^, it is one of the few scales with evidence of reliability and responsiveness, and it demonstrated good structural validity and internal consistency, which makes it a promising instrument. In the future, it is important to check the scale’s content validity on general population and investigate the floor effect. MAILS^42^ is also a promising instrument, with good evidence for structural validity, internal consistency, and construct validity. It is the only scale with evidence for minimal floor and ceiling effects. In the future, it is important to check the scale’s content validity on general population. AISES^31^ also showed good evidence for structural validity and internal consistency, but as with the previous two instruments, it is important to check the scale’s content validity on general population. Lastly, most SAIL4ALL^34^ subscales showed good evidence for structural validity and internal consistency, however, the psychometric properties of “What can AI do?” subscale are questionable. SAIL4ALL is currently the only available performance-based scale targeting general population.

When aiming for an assessment of higher education students, AI literacy test^32^ and ChatGPT literacy scale^38^ are the scales with the most robust quality evidence. Both showed at least some evidence for content validity while showing good evidence for structural validity, internal consistency, and construct validity. AI literacy test^32^ is the only performance-based scale available now targeting higher education students. MAIRS-MC^43^ also showed at least some evidence for content validity while showing good evidence for structural validity and internal consistency. GSE-6AI^30^, Hwang et al.’s instrument^39^, and Chan & Zhou’s EVT based instrument (knwl. of gen. AI subscale)^37^ are also promising instruments with good evidence for structural validity and internal consistency, however, their content validity needs to be checked on the higher-education students. GSE-6AI^30^, MAIRS-MC^43^, and SNAIL^45^ have been validated specifically for medical students, which makes them the instruments of choice if medical students are to be assessed.

When aiming for an assessment of secondary education students, AI-CI^33^, AILQ^35^ and Kim & Lee’s instrument^41^ all provided evidence for content validity, structural validity, and internal consistency, although AI-CI^33^ and AILQ^35^ had higher level of evidence for content validity and provided evidence for responsiveness. The decision between the two instruments might, to some degree, be guided by the languages they are available in, with AI-CI^33^ and AILQ^35^ currently available only in English, and Kim & Lee’s instrument^41^ only in Korean.

When aiming for an assessment of teachers’ perceived readiness to implement AI into their pedagogical practice, Intelligent TPACK^40^ in the only instrument available now. It showed good evidence for structural validity and internal consistency, however, its content validity needs to be checked on the teachers.

There are several general recommendations for future research. Cross-cultural validity, measurement error, and floor and ceiling effects of the existing scales should be checked. If the authors of the scales made the raw data open, it would solve many problems as, for example, multiple group factor analyses require raw data for comparison. With a single performance-based scale available^32^ targeting higher education students, it might be beneficial to design performance-based scales aimed at different populations as well. It would also be beneficial to cross-validate the results of the performance-based and self-report scales. Finally, it will be necessary to review the state of AI literacy scales in the future and update the current quality assessment.

This review has some limitations. It was performed by a single author, which might have caused some bias in the scales’ quality assessment, despite the COSMIN quality criteria being straightforwardly and quantitatively stated in the COSMIN manuals. Then, some AI literacy scales might have been missed if published in grey literature, since the search was limited to Scopus and arXiv. However, the chances of missing some relevant scales were reduced by the reversed search in Scopus and Google Scholar.

## Methods

To address the objectives of this study, I employed a systematic review followed by a quality assessment of AI literacy scales. I performed the review in accordance with the updated PRISMA 2020 guidelines^64^. The study was preregistered at OSF at https://osf.io/tcjaz.

### Literature search

I conducted the literature search on June 18, 2024, ensuring coverage of all literature available up to mid-2024. Initially, I conducted the search on January 1, 2024, as planned in the preregistration. However, due to the dynamically evolving field, I decided to redo the search during the first round of peer review to include the most up-to-date sources. I searched for literature in two databases—Scopus and arXiv. Scopus served as a primary database for peer-reviewed articles with arXiv supplementing Scopus with its coverage of pre-prints. I created search strings (Table 5) after an iterative process of finding and adding relevant terms and removing terms yielding irrelevant results^65^. I set no limits on publication date, publication type, or publication stage. In Scopus, I searched in titles, abstracts, and keywords; in arXiv, I searched in all fields. In Scopus, I limited the search to English papers. Additionally, in conjunction with the database searches, I looked for sources in reference lists of the included studies, as well as by a reversed search by works citing the included studies in Scopus and Google Scholar on June 20, 2024.

**Table 5:** Search strings

database	search string
Scopus	TITLE-ABS-KEY ((AI OR artificial*intelligence) AND (literacy OR skills OR knowledge) AND (scale OR test OR exam OR questionnaire OR survey)) AND (LIMIT-TO(LANGUAGE, "English"))
arXiv	(AI OR artificial*intelligence) AND (literacy OR skills OR knowledge) AND (scale OR test OR exam OR questionnaire OR survey)

### Inclusion criteria

Studies met the inclusion criteria if they: (1) developed new or revalidated existing AI literacy scale, (2) provided the full item list, (3) described how the items were formulated, (4) described the study participants, and (5) described validation techniques used in the scale development.

### Data extraction

I extracted the following data from the studies: name(s) of the author(s), date of the publication, scale type (self-report or performance-based), number and type of the items, language(s) that the scale is available in, target population, participant characteristics, factor extraction method, factor structure, and data related to the quality assessment procedure as described in the Quality assessment section. I emailed authors for information missing in the articles—often the age distributions of the participants—and, when available, I also used published datasets to compute the missing information. Most information on completion time, missing data, and floor and ceiling effects were calculated from the published datasets.

### Quality assessment

First, I evaluated methodological quality of the individual studies by using the *COnsensus‑based Standards for the selection of health Measurement INstruments* (COSMIN)^52–54^ for the self-report scales, and additionally the *COSMIN Risk of Bias tool to assess the quality of studies on reliability or measurement error of outcome measurement instruments*^55^ for the performance-based scales. While the COSMIN tool was originally devised for the medical field, it has since been used in both psychological^66,67^ and educational research^68^. The psychometric qualities of self-reports are generally consistent across these fields, making the COSMIN tool satisfactory for use in diverse research areas.

Drawing from the COSMIN tool, I assessed the scales based on the measurement properties of content validity, structural validity, internal consistency, cross-cultural validity, reliability, measurement error, construct validity, and responsiveness. I did not evaluate the scales based on the criterion validity as suggested in the COSMIN tool because as of January 2024, there was no gold standard tool for measuring AI literacy. I assessed each measurement property by a box containing several questions scored on the scale of *very good, adequate, doubtful*, and *inadequate*, according to the defined COSMIN criteria^56^. A system of worst score counts applied for each box. Additionally, I assessed the scales based on the criteria of interpretability and feasibility—while not being measurement properties, COSMIN recognizes them as important characteristics of the scales.

Then, I applied the criteria for good measurement properties by using COSMIN quality criteria for the individual studies. The criteria assess the measurement properties on a scale of *sufficient, insufficient*, and *indeterminate*. Studies assessed as *sufficient* on some measurement property had to report a given metrics and the metrics had to be above a quality threshold set by COSMIN. On the other hand, studies assessed as *insufficient* on some measurement property reported a given metrics, but the metrics was under the quality threshold set by COSMIN, while studies assessed as *indeterminate* on some measurement property did not report a given metrics.

Finally, I synthetized the evidence per measurement property per scale. I rated the overall results against the criteria for good measurement properties and used the Grading of Recommendations Assessment, Development, and Evaluation (GRADE) approach for systematic reviews of clinical trials^57^ to come to a final scale-level quality rating. In case of the scales which have been revalidated, I pooled the estimates from the individual studies with a random-effect meta-analysis in *R*^69^ package *metafor*^70^ and gave rating based on the pooled estimates. The individual methodological quality ratings as well as the quality criteria ratings with the COSMIN thresholds are available as Supplementary Data 1. Table 6 shows the interpretation of the overall levels of evidence for the quality of the measurement properties.

**Table 6:** Overall levels of evidence for the quality of the measurement properties

quality of evidence	rating	description
high	++++/−−−−	consistent findings in multiple studies of at least adequate quality OR in one study of very good quality
moderate	+++/−−−	consistent findings in multiple studies of at least doubtful quality OR in one study of adequate quality
low	++/−−	consistent findings in multiple studies of at least inadequate quality OR in one study of doubtful quality
very low	+/−	finding only from one study of doubtful quality
conflicting	±	conflicting findings
indeterminate	?	only studies of poor methodological quality

#### Content validity

Content validity refers to the degree to which the instrument measures the construct(s) it purports to measure^71^. COSMIN considers content validity the most important measurement property of an instrument as it should be ensured that the instrument is relevant, comprehensive, and comprehensible with respect to the construct of interest and study population^54^. COSMIN requires that both experts and target population are involved in content validation for content validity to be considered adequate.

#### Structural validity

Structural validity refers to the degree to which the instrument scores are an adequate reflection of the dimensionality of the construct to be measured. COSMIN requires that factor analyses or IRT/Rasch analyses are used to assess structural validity^71^.

#### Internal consistency

Internal consistency refers to the degree to which the items are interrelated. COSMIN requires Cronbach’s alpha(s) to be calculated for each unidimensional scale or subscale^71^.

#### Measurement invariance

Measurement invariance refers to the degree to which the factor structure remains same for various subgroups—i.e., gender, age, or level of education—and whether the items exhibit Differential Item Functioning (DIF). COSMIN requires multiple group factor analysis or DIF analysis to be used to assess measurement invariance^71^.

#### Cross-cultural validity

Cross-cultural validity refers to the degree to which the performance of the items on a translated or culturally adapted scale are an adequate reflection of the performance of the items of the original version of the scale. COSMIN requires multiple group factor analysis or DIF analysis to be used to assess cross-cultural validity^71^.

#### Reliability

Reliability refers to the proportion of total variance in the measurement which is because of true differences among participants. COSMIN requires reliability to be assessed by intra-class correlation coefficients or weighted Kappa and it requires multiple observations in time^71^.

#### Measurement error

Measurement error refers to the systematic and random error of participants’ scores which are not attributed to true changes in the construct to be measured. COSMIN requires smallest detectable change or limits of agreement to be measured to assess the measurement error. As with reliability, it requires multiple observations in time^71^.

#### Construct validity

Construct validity refers to the degree to which the scores are consistent with hypotheses based on the assumption that the scale validly measures the intended construct. COSMIN requires a comparison to either another scale aiming to measure a similar construct or hypothesis testing among subgroups^71^.

#### Responsiveness

Responsiveness refers to the scale’s ability to detect change over time in the construct to be measured. COSMIN allows several ways to test scale’s responsiveness including hypothesis testing before and after intervention, comparison between subgroups, comparison with other outcome measurement instruments, or comparison to a gold standard^71^.

#### Interpretability

Interpretability refers to the degree to which one can assign qualitative meaning to the scores or changes in scores^71^. I included an assessment of overall scores’ distributions, missing data, and floor and ceiling effects. Overall scores’ distributions show if the scale results in normally distributed data. Missing data should be minimized to ensure they did not affect the validation procedure. Finally, floor and ceiling effects show whether the extreme items are missing in the lower or upper end of the scale, indicating limited content validity. Consequently, participants with the lowest or highest possible score cannot be distinguished from each other, thus reliability is reduced. I considered floor and ceiling effects to be present if more than 15% of respondents achieved the lowest or highest possible score, respectively^72^.

#### Feasibility

Feasibility refers to the ease of application of the scale in its intended context of use, given constraints such as time or money^73^. I checked the languages in which the scales are available and the scales’ completion times.

### Supplementary information

PRISMA Checklist

Supplementary Data 1

## Data Availability

All data generated or analysed during this study are included in this published article.
